# Assessing patient engagement approaches in the development of patient reported outcome measures

**DOI:** 10.1186/s41687-026-01002-7

**Published:** 2026-01-29

**Authors:** Farheen Khan, Michelle Prunier, Ivana Ristevski, Maja Trantalovski, Helen Dimaras

**Affiliations:** 1 Department of Ophthalmology and Vision Sciences, The Hospital for Sick Children, 555 University Avenue, Toronto, Ontario M5G 1X8 Canada; 2 Institute of Medical Sciences, University of Toronto, Toronto, Ontario Canada; 3 Patient Partner, The Hospital for Sick Children, Toronto, Ontario Canada; 4 Department of Ophthalmology and Vision Sciences, University of Toronto, Toronto, Ontario Canada; 5 Child Health Evaluative Sciences Program, SickKids Research Institute, Toronto, Ontario Canada; 6 The Centre for Global Child Health, SickKids Research Institute, Toronto, Ontario Canada; 7 Division of Clinical Public Health, Dalla Lana School of Public Health, University of Toronto, Toronto, Ontario Canada

## Abstract

**Background:**

Guidelines for the development and validation of patient-reported outcome measures recommend incorporating patient input to ensure relevance and comprehension, but do not clearly define what patient input entails. As a result, meaningful patient engagement is often conflated with research participation. This study uses the adaptation and validation of the FACE-Q Craniofacial Module patient-reported outcome measure for ophthalmology patients as a case study to showcase how patients can be engaged as partners across the different phases of patient-reported outcome measure adaptation, and illustrate how their involvement influenced the adaptation process.

**Methods:**

Patient engagement strategies across five phases of the adaptation and validation of the FACE-Q Craniofacial Module (Study development, Item adaptation, Item reduction, Psychometric evaluation, and Dissemination) were retrospectively mapped to the International Association for Public Participation Spectrum. This framework outlines five levels of engagement: Inform, Consult, Involve, Collaborate, and Empower, and was used to evaluate the extent and nature of engagement.

**Results:**

Patient partners (*n* = 8) with lived experience of retinoblastoma, strabismus, corneal anesthesia, and ocular prostheses were engaged across the five phases of the study. Mapping engagement activities to the International Association for Public Participation Spectrum revealed that the “Inform” and “Involve” levels of engagement were present in all five phases. The most extensive engagement, spanning all levels of the International Association for Public Participation Spectrum, occurred during the “Item Reduction” and “Dissemination” phases of the study.

**Conclusion:**

The current case study showcases that patient engagement can be incorporated intentionally across all phases of patient-reported outcome measure adaptation and validation. The International Association for Public Participation Spectrum provided a structured approach to map and document the nature and extent of engagement across our study. Our case study may support the planning, execution, and reporting of patient engagement strategies in future patient-reported outcome measure development and adaptation studies.

## Background

Patient-reported outcome measures (PROMs) are critical for incorporating the patient voice into healthcare decision-making. PROM development guidelines broadly recommend patient input throughout PROM development and validation to ensure relevance and comprehension [1–3], but do not clearly define what constitutes “patient input”. As such, patient input has largely been interpreted as and limited to research participation [4]. As participants, patients provide data and have limited influence over decisions, which are made primarily by researchers [5]. However, patient input can extend beyond research participation and involve patient engagement, or meaningful collaboration between researchers and patients as partners in governance, priority setting, question development, and study execution [5]. Engaging patients while developing and validating PROMs can lead to more relevant and patient-centred measures through building trust, iterative refinement, and prolonged emphasis on patient-driven priorities [6].

Though there has been increased recognition in the importance of patient engagement in research [7], there is a significant gap in PROM studies [4]. A scoping review from 2016 [4] based on 189 studies spanning a wide range of clinical areas from 1980 to 2014, discovered that while approximately 75% of the PROMs involved patients as research participants during item development (58.5%) and testing draft or pilot PROMs for comprehensibility (50.8%), only 9.8% involved patients in the development of conceptual frameworks, with 10.9% having engaged patients in determining which outcomes to measure. Similar trends have been observed in more recent studies [8–11], in which patient “engagement” was found to be limited, and primarily restricted to research participation during item development. To guide and evaluate levels of patient engagement across study phases, research teams [7, 12] have adapted the International Association for Public Participation (IAP2) Spectrum [13], which outlines five levels of engagement, increasing by influence in decision-making: inform (providing information), consult (seeking feedback), involve (ensuring concerns are considered), collaborate (work together towards decisions), empower (patients make final decisions). However, even in studies applying the IAP2 Spectrum to categorize engagement, participation and engagement are often conflated. For example, in one study [14], co-creating items with youth or co-conducting research (e.g., researchers and youth both facilitating focus groups) were correctly categorized as “collaborate.” Conversely, interviews and focus groups, which would typically be considered research participation, were also categorized as “collaborate,” distorting the true scope of patient engagement.

To address these challenges, our research team embedded patient engagement throughout the process of adapting and validating the FACE-Q Craniofacial Module (FACE-Q) [15, 16], for ophthalmic patients (i.e., patients with strabismus, corneal anesthesia, retinoblastoma, and ocular prostheses). In this short report, we retrospectively map the patient engagement strategies used in our study to the IAP2 Spectrum to demonstrate how intentional and sustained patient engagement can be implemented in PROM development and validation. We also share our reflections to inform future patient engagement efforts in PROM generation, adaptation, and validation studies.

## Methods

### Study context

Given the patient-informed gaps in the availability of standardized tools to evaluate appearance and psychosocial impacts experienced by children and adults with strabismus [17], corneal anesthesia [18], retinoblastoma [19], and ocular prostheses [20], the FACE-Q was adapted to address this need. The FACE-Q is a modular PROM with independently functioning scales and checklists assessing appearance, function, adverse effects, and health-related quality of life (HRQoL) in patients with facial differences [15, 16]. It was rigorously developed and validated with patient input for patients aged 8–29, and is included in the ICHOM Standard Sets for craniofacial conditions [21]. The adaptation process included five phases: 1) study development; 2) item generation/adaptation with input from patients, clinicians, and scientists; 3) item reduction; 4) psychometric validation; and 5) dissemination.

The ‘Study Development’ phase involved preparing study materials, including participant-facing documents (e.g., consent forms, recruitment materials), interview guides for content validity assessment, and reminder guides for scheduled interviews or PROM completion during field testing. Research Ethics Board approval was also obtained during this phase (REB #1000079009). The ‘Item Generation/Adaptation’ phase involved amalgamating the data from two rounds of cognitive debriefing interviews with patient participants (pediatric and adult corneal anesthesia, retinoblastoma, and strabismus patients aged 8 years of age or older), which aimed to assess the content validity of the original FACE-Q for the ophthalmic conditions and identify the need for novel scales. After each round, these findings were discussed with an expert panel of ophthalmologists, scientists, and patient partners to guide necessary modifications. The “Item Reduction” phase involved recruiting a large sample size (200+ pediatric and adult corneal anesthesia, retinoblastoma, and strabismus patients aged 8 years of age or older) to field-test the pilot version of the modified FACE-Q for item reduction via Rasch analysis. The ‘Psychometric Validation’ phase involved the psychometric validation of the finalized and modified FACE-Q to ensure it functions as intended (construct validity via a priori hypotheses testing), is reliable (test-retest reliability using intraclass correlation coefficients), and sensitive to change in the patient populations of interest (responsiveness analyses using anchor-based methods). The final ‘Dissemination’ phase involved disseminating the results of the adaptation and validation of the modified FACE-Q in peer-reviewed journals, at conferences, and in community-facing events.

### Patient partner involvement

Patient partners were recruited from existing partners affiliated with our lab, clinician referrals, and patient advocacy groups. Patients were eligible to join the study team as patient partners if they had corneal anesthesia, retinoblastoma, or strabismus, and were 8 years of age or older. In the first two phases, patients participating in cognitive debriefing interviews were ineligible to be involved as patient partners. In the final two phases, patients participating in the field-testing process were ineligible, while cognitive debriefing interview participants were now eligible.

### Mapping to the IAP2 spectrum

Patient engagement strategies were retrospectively mapped to the IAP2 spectrum based on patient contributions in each study phase. In cases where activities reflected more than one IAP2 level, they were broken down to highlight the components associated with the different levels of engagement.

## Results

### Patient partner demographics

Patient partners included one “parent in research” affiliated with our lab [22], whose proximity allowed for more regular and readily available engagement throughout the study, as well as seven additional partners recruited through a retinoblastoma research advocacy group [23] (*n* = 4) and clinician referrals (*n* = 3). The patient partners represented a diverse range of experiences across the ophthalmic conditions of interest (one corneal anesthesia patient, two strabismus patients, and five retinoblastoma patients), included a teenager (*n* = 1), young adults (*n* = 4), and adults (*n* = 3), and mainly consisted of females (*n* = 7). Half of the patient partners were involved during the first two phases, while the remaining half joined the team during item reduction phase.

### Phase 1: Study development

**Fig. 1:**
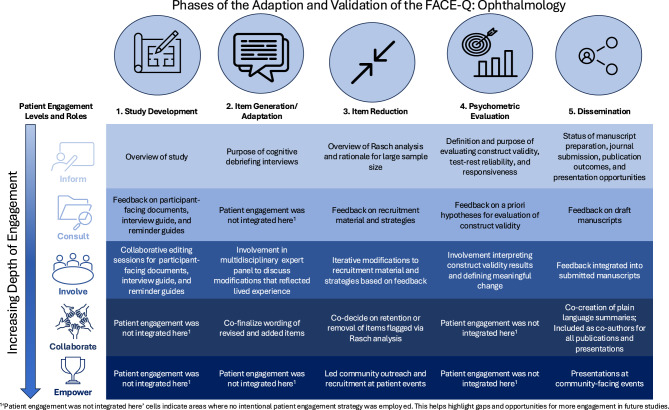
Mapping of the patient engagement strategies to the IAP2 Public engagement spectrum. Patient engagement strategies are depicted across the five phases of PROM adaptation and validation (horizontal) and the scope of our patient engagement strategies across the five levels of engagement (vertical). The “inform” and “involve” levels were most consistently operationalized, spanning all five phases of our study, and the “empower” level was least operationalized, incorporated only in the final phase. The “item reduction” and “dissemination” phases encompassed all five levels of patient engagement during the adaptation and validation of the modified FACE-Q

In this phase, patient engagement spanned the inform, consult, and involve levels of the IAP2 spectrum (Fig. 1). The collaborate and empower levels were not applied. The patient partners were informed of the study through sharing the sharing the original FACE-Q and our study protocol, as well as providing verbal overviews of the project, ensuring space was held for questions. We then consulted with patient partners on all study materials through collaborative editing sessions (involve), which ensured clarity, accessibility, and sensitivity through co-learning and rich discussions.

With patient engagement, our recruitment materials were revised to be accessible for individuals with visual impairments (e.g., improved colour contrast, incorporation of alt text). Further, due to the psychosocial complexity of our target populations and appearance-based concepts, the language in all our study documents were tailored to be mindful and respectful of the sensitive-nature of patients. Input from patient partners also ensured that the participant-facing documents were easy to navigate, interview guides were comprehensive, and reminder guides were empathetic. Early engagement built strong rapport between the patient partners and the research team, and established a sense of shared ownership, which laid the foundation for meaningful engagement and collaboration in the subsequent phases of the study.

### Phase 2: Item generation/adaptation [24]

Here, patient engagement spanned the inform, involve, and collaborate levels (Fig. 1). The consult and empower levels were not applied. Prior to each expert panel discussion, the study team informed stakeholders of the purpose of the cognitive debriefing interviews (i.e., evaluate the content validity of the FACE-Q and modify as necessary). During the discussions, the perspective of the patient partners was prioritized and revisited when reaching consensus to ensure that all content modifications reflected the lived experiences of patients (involve). After the panel discussions, the study team further collaborated with the patient partners to finalize the wording of revised or new items.

Patient engagement in this phase was imperative to ground the adaptation process in patient experience, rather than relying solely on clinical and research expertise. Patient partners helped clarify ambiguities from the cognitive interviews by drawing on both their personal experiences and those of patients in their communities, strengthening the content validity of the adapted and modified FACE-Q. For example, they emphasized the need for a novel scale to evaluate the appearance of ocular prostheses, which was subsequently co-developed with input from both patient partners and research participants. The patient partners also ensured that the content modifications reflected what the research participants truly meant, in clear, inclusive language.

### Phase 3: Item reduction

Patient engagement spanned all five levels in this phase (Fig. 1). First, the study team informed the patient partners of the rationale for a large sample size and explained what Rasch analysis is through an accessible and simplified presentation. Then, quarterly working group meetings were held to co-develop recruitment materials and discuss recruitment progress and strategies. Feedback was incorporated by the study team both during and after the discussions and shared at subsequent meetings (consult and involve). Patient partners were also empowered to share information about the study and its importance in patient community settings. Once Rasch analysis was complete, the two best iterations of each scale were presented to an expert panel of ophthalmologists, scientists, and patient partners to discuss the removal or retainment of flagged items (collaborate).

In terms of recruitment, patient engagement led to a more patient-centred approach, where the emphasis by patient partners on attention-grabbing designs, impactful language on recruitment material, and participant compensation, as well as their leadership within their communities, ultimately strengthened our recruitment efforts. By embedding patient engagement into the item reduction process, we assured that the final selection of items comprising each scale in the modified FACE-Q reflected both, good measurement properties and the lived experience.

### Phase 4: Psychometric validation

In this phase, patient engagement spanned the inform, consult, and involve levels (Fig. 1). The collaborate and empower levels were not applied. The study team first informed the patient partners in plain language the definitions and purpose of evaluating construct validity, test-retest reliability, and responsiveness. The a priori hypotheses were developed based on existing literature by the study team and then shared with the patient partners for feedback and input on additional hypotheses (consult). The construct validity and responsiveness results were discussed with the patient partners to include the patient perspective in interpreting the results and defining meaningful change (involve).

Similar to phase 3, patient engagement was essential to the evaluation of the psychometric properties of the finalized and modified FACE-Q to ensure that the statistical outcomes pertaining to construct validity and responsiveness aligned with patients’ lived experiences. Patient engagement in this phase highlighted the value of including the patient perspective, even when complex psychometric concepts are involved, to foster shared understanding and trust in the results, and ensure that the final PROM is both psychometrically sound and patient-centred.

### Phase 5: Dissemination

Patient engagement spanned all five levels in this phase (Fig. 1). All patient partners were informed of the status of manuscript preparation, submission to journals, publication outcomes, and presentation opportunities. Draft manuscripts were shared with the patient partners feedback (consult) and editing (involve). Plain language summaries were co-authored with the patient partners and all patient partners were listed as co-authors across manuscripts, posters, and oral presentations (collaborate). Patient partners also shared their experiences and impact as partners in research through oral conference presentations and blog posts at events and on websites hosted by patient advocacy groups respectively (empower).

The leadership of the patient partners in community-facing activities provided them the opportunity to amplify patient voices and expand research dissemination beyond just reporting results, strengthening the accessibility of dissemination outputs beyond academic and clinical communities. For example, at a research seminar for retinoblastoma survivors and families, one patient partner provided a talk about their experience on the project and the impact of developing such a tool rooted in patient priorities. By sharing their own narratives about the research process, they highlighted the true purpose of patient engagement: ensuring that research reflects, empowers, and reaches the patient communities it aims to serve.

## Discussion

Through the application of the IAP2 spectrum, we assessed the scope and depth of our patient engagement strategies across five phases of PROM adaptation and validation, offering a structured lens to highlight strengths and areas for growth. In our study, the “inform” and “involve” levels were the most feasible and consistently applied. While portrayed as lower levels of engagement on the spectrum, they laid the foundation for strong rapport and sustained relationships, aligned with the readiness of patient partners and the constraints of our study timelines and resources. Successful engagement in these levels, particularly in the earlier phases of the study, coupled with the fact that this study was built on patient-identified priorities, enabled the progression towards deeper engagement in subsequent phases. Notably, while the structured and technical nature of PROM development and validation may appear as a barrier for shared decision-making via collaboration with and empowerment of patient partners, in our case, patient engagement spanned all five levels during the measurement-heavy item reduction phase.

Mapping our patient engagement strategies to the IAP2 spectrum helped surface these strengths and revealed areas where additional patient engagement strategies could be considered for future studies. In addition, our early and continuous involvement of patient partners, spanning from the development of study materials all the way through to dissemination efforts, complemented the data we collected from patients as research participants. This ensured that the lived experience of patients was reflected across the full adaptation and validation of the modified FACE-Q

Broadly, the IAP2 spectrum provided a helpful framework for retrospective reflection and prospective planning of patient engagement strategies, as well as distinguishing patient engagement from patient involvement as research participants – an area where the broader PROM development literature has noted conceptual overlap. Although our case was not designed to link engagement levels to PROM performance or compare the effects of different engagement strategies, future studies may use this framework to examine how and whether varying levels of engagement influence PROM psychometric properties, and may also draw on our example to categorize patient engagement in PROM development more transparently by differentiating it from research participation.

## Data Availability

Data sharing is not applicable to this article as no datasets were generated or analysed during the current study.
